# Genomic Analysis of Vancomycin-Resistant Staphylococcus aureus Isolates from the 3rd Case Identified in the United States Reveals Chromosomal Integration of the *vanA* Locus

**DOI:** 10.1128/spectrum.04317-22

**Published:** 2023-03-28

**Authors:** Wolfgang Haas, Navjot Singh, William Lainhart, Lisa Mingle, Elizabeth Nazarian, Kara Mitchell, Geetha Nattanmai, Donna Kohlerschmidt, Michelle C. Dickinson, Marilyn Kacica, Nellie Dumas, Kimberlee A. Musser

**Affiliations:** a Wadsworth Center, New York State Department of Health, Albany, New York, USA; University of West London

**Keywords:** vancomycin-resistant *Staphylococcus aureus*, VRSA, antibiotic resistance, *Staphylococcus aureus*, vancomycin, whole-genome sequencing

## Abstract

Vancomycin-resistant Staphylococcus aureus (VRSA) is a human pathogen of significant public health concern. Although the genome sequences of individual VRSA isolates have been published over the years, very little is known about the genetic changes of VRSA within a patient over time. A total of 11 VRSA, 3 vancomycin-resistant enterococci (VRE), and 4 methicillin-resistant S. aureus (MRSA) isolates, collected over a period of 4.5 months in 2004 from a patient in a long-term-care facility in New York State, were sequenced. A combination of long- and short-read sequencing technologies was used to obtain closed assemblies for chromosomes and plasmids. Our results indicate that a VRSA isolate emerged as the result of the transfer of a multidrug resistance plasmid from a coinfecting VRE to an MRSA isolate. The plasmid then integrated into the chromosome via homologous recombination mediated between two regions derived from remnants of transposon Tn*5405*. Once integrated, the plasmid underwent further reorganization in one isolate, while two others lost the staphylococcal cassette chromosome *mec* element (SCC*mec*) determinant that confers methicillin-resistance. The results presented here explain how a few recombination events can lead to multiple pulsed-field gel electrophoresis (PFGE) patterns that could be mistaken for vastly different strains. A *vanA* gene cluster that is located on a multidrug resistance plasmid that is integrated into the chromosome could result in the continuous propagation of resistance, even in the absence of selective pressure from antibiotics. The genome comparison presented here sheds light on the emergence and evolution of VRSA within a single patient that will enhance our understanding VRSA genetics.

**IMPORTANCE** High-level vancomycin-resistant Staphylococcus aureus (VRSA) began to emerge in the United States in 2002 and has since then been reported worldwide. Our study reports the closed genome sequences of multiple VRSA isolates obtained in 2004 from a single patient in New York State. Our results show that the *vanA* resistance locus is located on a mosaic plasmid that confers resistance to multiple antibiotics. In some isolates, this plasmid integrated into the chromosome via homologous recombination between two *ant(6)-sat4-aph(3′)* antibiotic resistance loci. This is, to our knowledge, the first report of a chromosomal *vanA* locus in VRSA; the effect of this integration event on MIC values and plasmid stability in the absence of antibiotic selection remains poorly understood. These findings highlight the need for a better understanding of the genetics of the *vanA* locus and plasmid maintenance in S. aureus to address the increase of vancomycin resistance in the health care setting.

## INTRODUCTION

Until the emergence of vancomycin-resistant enterococci (VRE) and Staphylococcus aureus (VRSA) in 1986 and in 2002, respectively, the glycopeptide vancomycin was one of the few antibiotics remaining to treat infections caused by multidrug-resistant strains of these pathogens (1, 2). Vancomycin inhibits cell wall synthesis in Gram-positive bacteria by binding to and blocking d-Ala-d-Ala-peptidoglycan precursors from becoming incorporated into the growing cell wall (3). High-level vancomycin resistance is mediated by enzymes that modify the drug’s target such that the antibiotic can no longer bind efficiently. In the case of VanA-mediated resistance, VanH and VanA synthesize and incorporate new d-Ala-d-Lac precursors into the cell wall, while VanX hydrolyzes any existing d-Ala-d-Ala-peptidoglycan precursors (3–5). The entire *vanA* operon consists of the *vanRSHAXYZ* genes, which includes additional enzymes (*vanY*, *vanZ*) and regulatory genes (*vanR*, *vanS*) (3). The *vanA* locus is associated with the transposon Tn*1546*, which can transpose among different plasmids and has been credited with the dissemination of *vanA* among Enterococcus faecium and other enterococci (6). Transposition could be one reason for the variability of pulsed-field gel electrophoresis (PFGE) patterns observed among sequential VRE isolates from long-term care patients (7). Tn*1546* is frequently located on conjugative plasmids that can transfer from a donor to a recipient cell and even cross species barriers (8–10).

In 2002, the first high-level VRSA in the United States was isolated from a patient in Michigan. Genetic evidence indicated that a conjugative plasmid had been transferred from a VRE to an MRSA strain that cocolonized the same patient. The Tn*1546* element then transposed from the enterococcal to a staphylococcal plasmid, which allowed for its continued maintenance inside the new host (2).

In 2004, a VRSA strain was isolated from the urine culture of a patient in a long-term-care facility in New York State (11), which was epidemiologically unrelated to previous VRSA isolates from Michigan and Pennsylvania. The patient had a history of multiple sclerosis, diabetes, chronic heart failure, and renal insufficiency; she was nonambulatory and had the same roommate for years. The patient had been treated repeatedly for chronic urinary tract infections with various antibiotics, including amoxicillin, levofloxacin, linezolid, imipenem, gentamicin, azithromycin, and vancomycin.

Over the course of 4.5 months, bacterial isolates were collected from the patient, her roommate, and staff at the nursing home. Comprehensive testing was performed at the Wadsworth Center Bacteriology Laboratory to characterize all VRSA, methicillin-resistant Staphylococcus aureus (MRSA), and VRE isolates. A subset of individual isolates and a section of the patient’s nephrostomy tube were sent to the CDC for further analysis. Weigel et al. later described multiple isolates, including S. aureus, E. faecium, and Enterococcus faecalis, obtained during the initial 4-week period from the urine, rectum, and a polymicrobial biofilm taken from the patient’s nephrostomy tube (12). Several of the S. aureus isolates were VRSA isolates (including two from urine and one from the biofilm) that were resistant to multiple antibiotics. The *vanA* gene was found on a plasmid that was present in the S. aureus urine isolate VRSA595 and the E. faecium biofilm isolate VRE2547. Notably, while isolate VRSA595 reverted to a vancomycin-susceptible phenotype on nonselective medium, a second VRSA isolate, VRSA5734, remained vancomycin resistant even after 20 subcultures (12). Pulsed-field gel electrophoresis (PFGE) typing of VRSA isolates from the nephrostomy tube distinguished four variants, all of which were of PFGE type USA800 (12).

Of the 14 VRSA that have been isolated in the United States to date, the vast majority are PFGE type USA100, which, together with USA800, comprise the clonal complex 5 (CC5) (13). CC5 is a widespread group of MRSA strains that is frequently associated with hospital-acquired infections (14). However, VRSA isolates are not limited to CC5 since the 13th VRSA isolate in the United States is PFGE type 1100 and CC30, which belongs to a group of community-acquired MRSA (15). A similarly diverse picture emerges with respect to the VRE that serve as *vanA* donors: while most VRE that have been identified as the source of vancomycin resistance in S. aureus are E. faecalis, other species, such as E. faecium, have been described as well (13, 15).

Previous whole-genome sequencing (WGS) studies that included two VRSA isolates from the New York (NY) patient showed differences in the presence of some of the antibiotic resistance genes, such as *aac(6′)-aph(2″)*, *ant(6)-Ia*, *aph(3′)-III*, and *tet*(S), but were unable to explain why the vancomycin resistance phenotype was stable in one isolate but not the other (16, 17). Since these studies included only one isolate per patient and did not produce closed genome assemblies, they were unable to show if and how the genomes changed over time. To gain a better understanding of the genetics underlying vancomycin resistance in S. aureus over a period of 4.5 months, we used a combination of long- and short-read sequencing to reconstruct the genomes of 18 clinical isolates obtained from the patient’s urine, rectum, and nephrostomy tube and one isolate from the patient’s roommate’s nares. Although the isolates were obtained from the same patient as those described in the Weigel study, they are not the same isolates (12). In the present study, one vancomycin-susceptible Enterococcus faecalis (VSE), three VRE, four MRSA, two vancomycin-resistant and methicillin-susceptible S. aureus (VRMSSA), and nine vancomycin- and methicillin-resistant S. aureus (VMRSA) isolates underwent a detailed sequence analysis. The latter two groups will be jointly referred to as VRSA in this report.

Our results indicate that a VRSA isolate emerged as the result of the transfer of a multidrug resistance plasmid from a coinfecting VRE to a MRSA isolate. The plasmid then integrated into the chromosome via homologous recombination mediated between two regions derived from remnants of transposon Tn*5405*. Once integrated, the plasmid underwent further reorganization in one isolate, while two others lost the staphylococcal cassette chromosome *mec* element (SCC*mec*) determinant that confers methicillin resistance. Variant calling showed that all 11 VRSA from the patient differed only by a few single-nucleotide polymorphisms (SNPs), indicating that they are all closely related. The results presented here explain how a few recombination events can lead to multiple PFGE patterns that could be mistaken for vastly different strains. A *vanA* gene cluster that is located on a multidrug resistance plasmid that is integrated into the chromosome could result in the continuous propagation of resistance, even in the absence of selective pressure from antibiotics.

## RESULTS

### The VRSA isolate was firmly established in the primary patient but did not spread to other patients or staff in the long-term-care facility.

A total of 650 specimens were collected in 2004 from the patient, her roommate, and facility staff as part of this investigation. The only vancomycin-resistant S. aureus isolates obtained were from the patient, which include the two methicillin-susceptible and nine methicillin-resistant isolates shown in Table 1. A MRSA isolate collected from the patient’s roommate, MRSA-WC000, was included in the present study for comparison.

**TABLE 1:** Characterization of bacterial isolates from New York State VRSA patient and her roommate

Isolate	Species	Collection date	Source	Real-time PCR	Vancomycin Etest MIC (μg/mL) and interpretation^*d*^	PFGE pattern^*e*^
VRE-WC031	E. faecalis	2 April 2004	Rectum	*vanA*	>256 R	
VSE-WC032	E. faecalis	2 April 2004	Rectum		1.5 S	
VRE-WC072	E. faecium	20 April 2004	Urine^*a*^	*vanA*	>256 R	
VRE-WC084	E. faecium	20 April 2004	Urine^*b*^	*vanA*	>256 R	
MRSA-WC000^*c*^	S. aureus	2 April 2004	Nares	*mecA*	NT	
MRSA-WC061	S. aureus	20 April 2004	Nephrostomy	*mecA*	NT	
MRSA-WC090	S. aureus	20 April 2004	Nephrostomy	*mecA*	NT	
MRSA-WC101	S. aureus	24 June 2004	Urine^*b*^	*mecA*	NT	
VMRSA-WC052	S. aureus	2 April 2004	Urine	*mecA*/*vanA*	128 R	C
VMRSA-WC062	S. aureus	20 April 2004	Nephrostomy	*mecA*/*vanA*	>32 R	D
VMRSA-WC071	S. aureus	20 April 2004	Urine^*a*^	*mecA*/*vanA*	32 R	D
VMRSA-WC081	S. aureus	20 April 2004	Urine^*b*^	*mecA*/*vanA*	16 R	D
VMRSA-WC082	S. aureus	20 April 2004	Urine^*b*^	*mecA*/*vanA*	6 I	F
VMRSA-WC083	S. aureus	20 April 2004	Urine^*b*^	*mecA*/*vanA*	>256 R	C
VMRSA-WC102	S. aureus	24 June 2004	Urine^*b*^	*mecA*/*vanA*	>256 R	E
VRMSSA-WC111	S. aureus	24 June 2004	Urine^*a*^	*vanA*	3 I	G
VRMSSA-WC113	S. aureus	24 June 2004	Urine^*a*^	*vanA*	32 R	H
VMRSA-WC121	S. aureus	18 August 2004	Urine	*mecA*/*vanA*	192 R	D
VMRSA-WC123	S. aureus	18 August 2004	Urine	*mecA*/*vanA*	192 R	D

a Straight catheter.

b Nephrostomy.

c Isolate from the roommate.

d CLSI Classification: ≤2 μg/mL, susceptible (S); 4 to 8 μg/mL, intermediate (I); ≥16 μg/mL, resistant (R); NT, not tested.

e All isolates were of PFGE type USA800. Letters C through H are in-house designations for different patterns.

The patient’s last vancomycin exposure had been in October of 2003. Despite this, and repeated antibiotic treatment, the patient remained VRSA-positive until at least 18 August 2004. Characterization of bacterial isolates from the patient included species identification, real-time PCR, and vancomycin Etest (Table 1). PFGE analysis grouped the 11 VRSA strains into 6 different SmaI restriction patterns.

### Hybrid genome assemblies demonstrate that the *vanA* locus is present on the chromosome in some VRSA isolates.

WGS was used to provide additional insights into the genetics underlying vancomycin resistance in these VRSA isolates. Short Illumina and long MinION reads were subjected to hybrid assembly with Unicycler (Table 2). The program produced circular assemblies for all DNA molecules in all isolates, except for two linear contigs of 52 and 216 kb in isolate VRE-WC084. These likely represent one or two plasmids that could not be circularized by the Unicycler program.

**TABLE 2:**
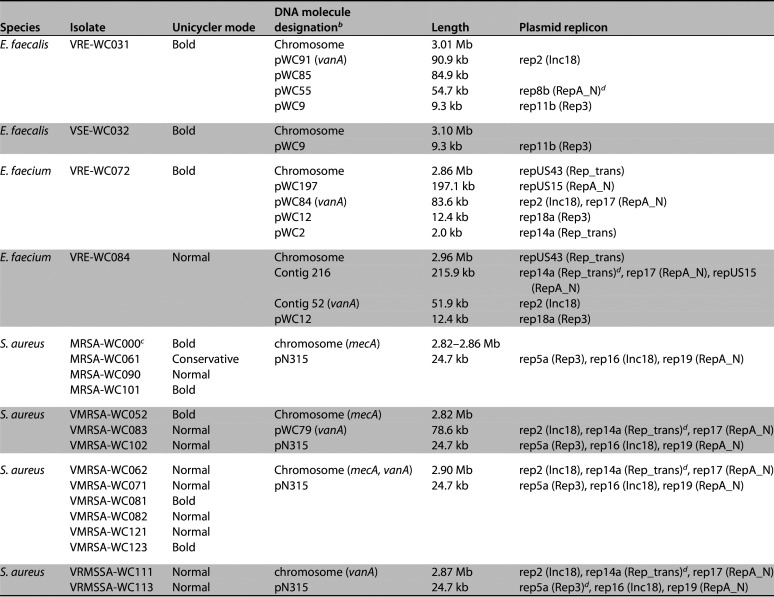
Results of the Unicycler hybrid genome assembly and plasmid replicon detection^*a*^

a Hybrid assembly with Unicycler was done using the settings “conservative,” “normal,” and “bold.” The mode that produced the best results for each isolate is shown.

b (*mecA*) and (*vanA*) indicate the location of these loci, where applicable.

c Patient’s roommate.

d Denotes a pseudogene.

The four enterococcal isolates contained between one and four plasmids, ranging in size from 2 to 197 kb. Among the VRE isolates, plasmid pWC84 of E. faecium VRE-WC072 contained the *vanA* locus and two plasmid replicons: rep17 (RepA_N) and rep2 (Inc18). Plasmid pWC91 from VRE-WC031 and contig 52 from VRE-WC084 also harbored the *vanA* locus and rep2 (Inc18) but lacked rep17 (RepA_N). All S. aureus isolates, regardless of phenotype, carried a 24.7 kb plasmid that shares 99.9% sequence identity with plasmid pN315 (18, 19). VRSA strains also possessed a second plasmid of 78.6 kb, here referred to as pWC79. PlasmidFinder identified three replicon sequences in pWC79: rep17 (RepA_N), rep2 (Inc18), and rep14a (Rep_trans), although the latter was interrupted by an IS*6* family transposase in all isolates (Table 2).

The hybrid assembly of long and short reads also revealed what *de novo* assembly using short reads only had failed to detect: pWC79 was present as a free plasmid in three of the VRSA isolates but integrated into the chromosome in the remaining eight VRSA isolates.

### Mobile genetic elements make the patient’s VRSA and VRE isolates highly multidrug resistant.

A search for antibiotic resistance genes revealed that the E. faecalis isolates VRE-WC031 and VSE-WC032 both carry the *erm*(B), *ant(6)-Ia*, and *aph(3′)-IIIa* genes, which confer resistance to macrolides, streptomycin, and amikacin/kanamycin, respectively (Table 3). While these three genes are located on the chromosome in VSE-WC032, they are present on plasmid pWC85 in VRE-WC031. VRE-WC031 also harbors a 90.9 kb plasmid, pWC91, that carries *vanA* as part of the *vanRSHAXYZ* locus, which confers resistance to vancomycin. The same plasmid also contains a copy of Tn*4001*, which is a composite transposon consisting of two genes, a *N*-acetyltransferase and a bifunctional *aac(6′)-aph(2″)* gene, that are flanked by IS*256* elements on either side (20). While the *aac(6′)-aph(2″)* gene is generally involved in aminoglycoside resistance, it is an inactive pseudogene in this and all other isolates, except for VMRSA-WC062, VMRSA-WC082, VMRSA-WC102, VMRSA-WC123, and VRSA-WC113.

**TABLE 3:**
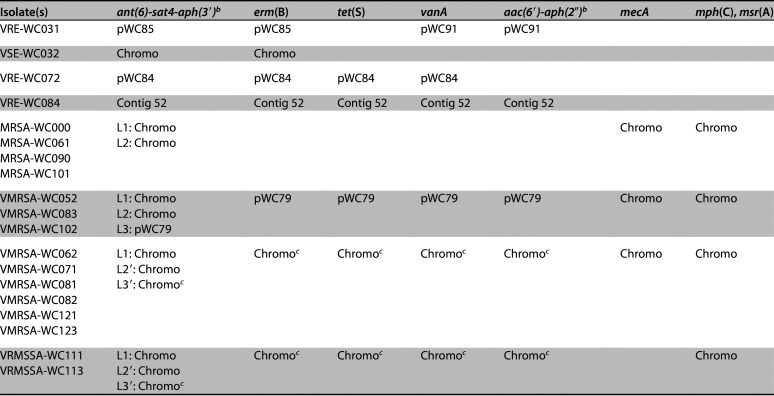
Location of select antibiotic resistance genes on the chromosome (chromo), plasmids, or contigs^*a*^

a Plasmid pWC79 can occur as a free plasmid or integrated into the chromosome. Two *ant*(6)-*sat*4-*aph*(3′)-*III* loci, L1 and L2, are located on the chromosome of all S. aureus isolates and a third, L3, on pWC79. L2′ and L3′ denote the corresponding loci after pWC79 integration into the chromosome.

b Some genes, such as *ant(6)-Ia*, *aph(3′)-IIIa*, or *aac(6′)-aph(2″)* might be only pseudogenes in some isolates or loci.

c The gene is located on the chromosome due to the chromosomal integration of plasmid pWC79.

In E. faecium VRE-WC72, an 83.6 kb plasmid, pWC84, carries the *ant(6)-Ia*, *sat4*, and *aph(3′)-IIIa* genes in addition to the *erm*(B), *vanA*, and *tet*(S) genes. The *sat4* and *tet(S)* gene products confer resistance to streptothricin and tetracycline, respectively. In E. faecium VRE-WC084, contig 52 carries the *ant(6)-Ia*, *sat4*, *aph(3′)-IIIa*, *erm*(B), *vanA*, and *tet*(S) genes as described for pWC84. In addition, contig 52 also carries the *aac(6′)-aph(2″)* pseudogene, a feature it has in common with pWC91 from VRE-WC031.

The chromosomes of all S. aureus isolates carry the *mph*(C) and *msr*(A) genes, conferring resistance to macrolides, and two copies of the *ant*(6)-*sat*4-*aph*(3′) locus, referred to as L1 and L2 from here on. Except for VRMSSA-WC111 and VRMSSA-WC113, which are methicillin susceptible, all S. aureus isolates possess the *mecA* gene, which confers methicillin resistance. The *blaZ* beta-lactam resistance gene, which is located on plasmid pN315, is present in all S. aureus isolates in this study.

**FIG 1:**
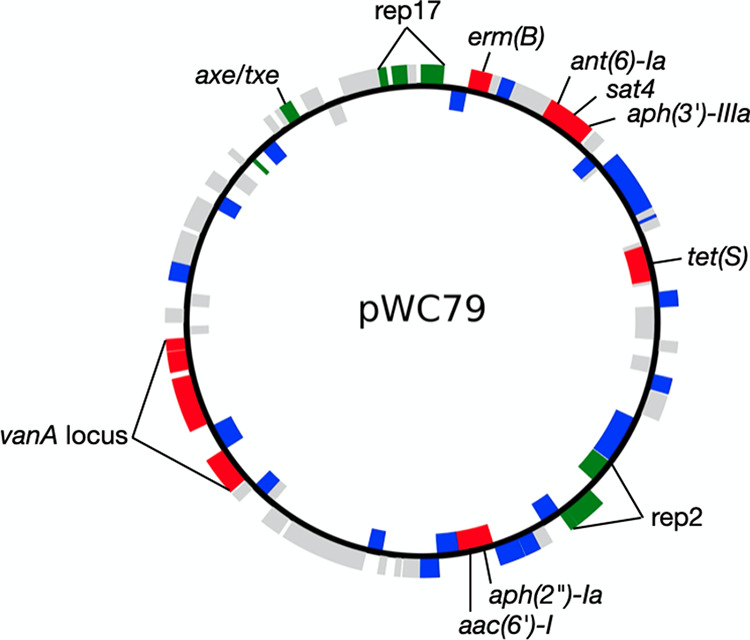
Schematic representation of the 78.6 kb plasmid pWC79. A more detailed view of the *vanA* locus is shown in Fig. 2. Outer ring, genes in the sense orientation; inner ring, genes in antisense orientation; red, antibiotic resistance genes; blue, transposase genes; green, replicons and the *axe*/*txe* toxin antitoxin system; gray, other genes.

All VRSA isolates possess a copy of plasmid pWC79 (Fig. 1, Table 3), which carries the *vanA* locus in addition to *erm*(B), *tet*(S), and *aac(6′)-aph(2″)*, the latter being a pseudogene in most isolates as described above. Plasmid pWC79 also carries a copy of the *ant(6)-sat4-aph(3′)* locus, designated L3. Unlike the two chromosomal loci L1 and L2, locus L3 includes an intact copy of the *ant(6)-Ia* gene.

In three of the isolates, VMRSA-WC052, VMRSA-WC083, and VMRSA-WC102, pWC79 is circular, which is consistent with an independently replicating plasmid. In the remaining VRSA isolates, pWC79 is integrated into the chromosome (Table 3).

### The Tn*1546* element that carries the *vanA* locus in the VRSA and VRE lacks the genes for transposition.

**FIG 2:**
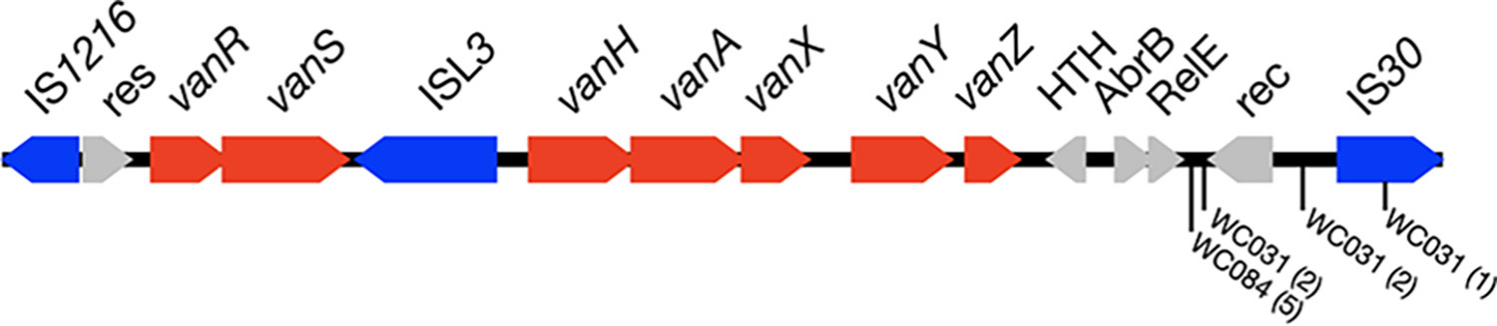
Schematic representation of the *vanA* locus and adjacent region in VRSA and VRE isolates (e.g., position 48,466 to 61,950 in pWC79 from VMRSA-WC052, CP092559). The left inverted repeat, transposase, and resolvase (*res*, ORF2 in Arthur et al. [6]) of Tn*1546* has been replaced by an IS*6*-like element IS*1216* family transposase. As in Tn*1546*, the locus includes the *vanRSHAXYZ* genes and the right inverted repeat, but unlike Tn*1546*, the locus also includes an ISL*3* family transposase. The locus is identical in all VRSA isolates and in VRE-WC072, while VRE-WC031 and VRE-084 differ by 5 SNPs each (indicated by the isolate name and the number of SNPs in parentheses). HTH, helix-turn-helix transcriptional regulator; AbrB, AbrB family transcriptional regulator; RelE, type II toxin-antitoxin system RelE/ParE family toxin; rec, recombinase family protein.

A comparison of the 9.8 kb region that includes the *vanA* operon among the different VRE and VRSA isolates shows that all share the same locus, regardless of species. Compared to Tn*1546* (6), the left inverted repeat, the transposase, and part of the resolvase has been replaced in all isolates by an IS*1216* family transposase (Fig. 2). The missing features were found nowhere else in the VMRSA-WC052 genome, suggesting that they are permanently lost. Another difference between the *vanA* locus in Tn*1546* and the *vanA* loci described in this study is that in all 14 VRE and VRSA isolates, the *vanRSHAXYZ* locus is interrupted by an ISL*3* family transposase that inserted between the *vanS* and *vanH* genes. This transposase is similar to the IS*1251* transposase located in the intergenic region between *vanS* and *vanH* in some E. faecium isolates (21) but differs from it by seven amino acids.

Aligning the *vanA* loci with progressiveMauve (22) showed 100% nucleotide identity for the 9.8 kb sequence (Fig. 2) in all VRSA and VRE isolates. However, two isolates stood out from the rest when five additional genes located 3′ to *vanZ* were included in the alignment. The region in VRE-WC031 differed by 5 SNPs and 10 indels from all other isolates, while 5 SNPs and 7 indels made the region in isolate VRE-WC084 unique (Fig. 2). Since the 13.5 kb region is identical in VRE-WC072 and all VRSA isolates, it makes this E. faecium isolate the most plausible source for transmitting *vanA* to S. aureus.

### The mosaic, multidrug resistance plasmid pWC79 from VRSA shares sequence elements with plasmids from Enterococcus faecium and Enterococcus faecalis.

Previous reports found that the *vanA* resistance locus was transferred via the Tn*1546* transposon from an enterococcal plasmid to a staphylococcal plasmid (2). In the present study, Tn*1546* lacks the genes for transposition, and we did not detect any staphylococcal plasmids that could have served as a recipient for Tn*1546* or the *vanA* locus. Strains VRE-WC031, VRE-WC072, and VRE-WC084 carry various plasmids or contigs that include replicons and genes that are also found in pWC79; however, none of the enterococcal isolates available for analysis harbor a copy of pWC79.

**FIG 3:**
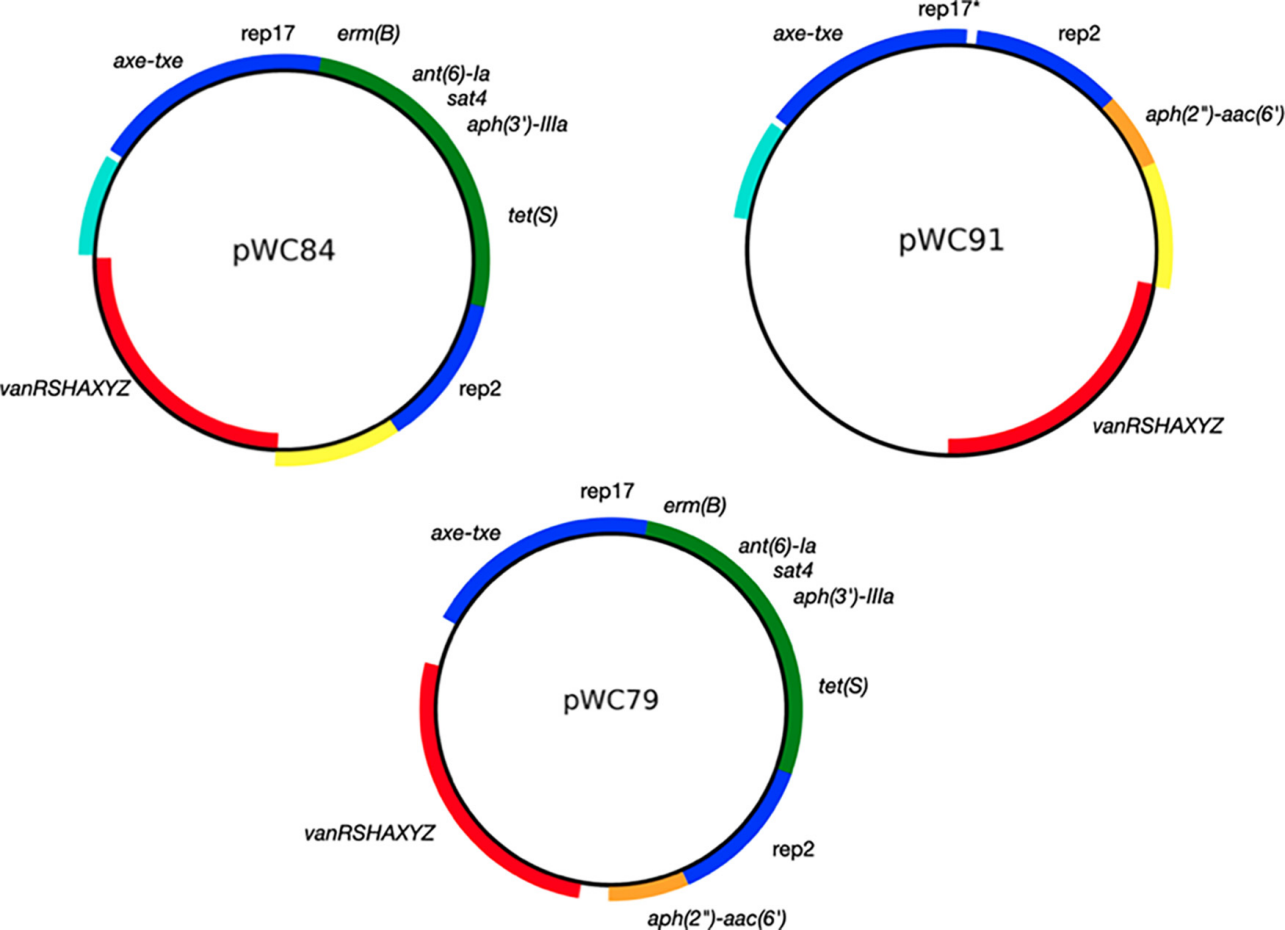
Comparison between pWC79 (78.6 kb) from VMRSA-WC052, pWC91 (90.9 kb) from VRE-WC031, and pWC84 (83.6 kb) from VRE-WC072. Regions of the same color are shared among the plasmids; regions that are depicted inside the plasmid are in the antisense orientation relative to pWC79. Genes of interest are indicated for reference; an asterisk (*) denotes that the gene is a pseudogene.

Plasmid pWC84 from E. faecium VRE-WC072 has a *vanA* locus identical to that found in pWC79 and has an almost identical complement of antibiotic resistance genes, making it the most likely predecessor of pWC79 (Fig. 3). However, pWC84 lacks the Tn*4001* transposon with the *aac(6′)-aph(2″)* gene. E. faecalis VRE-WC031 contains two plasmids of interest: pWC85 carries the *ant(6)-Ia*, *sat4*, *aph(3′)-IIIa*, and *erm(B)* genes (Table 3), while pWC91 carries a unique *vanA* locus and a copy of the *aac(6′)-aph(2″)* gene that is also found in pWC79. All three plasmids, pWC79, pWC84, and pWC91, have regions of DNA in common, such as rep2, rep17 (which is a pseudogene in pWC91), and the *axe/txe* locus. In addition, pWC84 (E. faecium) and pWC91 (E. faecalis) share two regions of DNA that are absent from pWC79.

These findings support the notion that pWC79 is a mosaic plasmid with origins in at least two of the VRE isolates, VRE-WC031 and VRE-WC072. This hypothesis is further supported by the high prevalence of insertion sequences and transposons in pWC79: 23 (24%) of the 96 genes in pWC79 encode transposases (Fig. 1), including ten IS*1216* family transposases, two IS*256* family transposases, one IS*1182* family transposase, one ISL*3* family transposase, and two IS*30* family transposases. The ability of these mobile genetic elements (MGEs) to mediate recombination events has been well documented (23) and will be described in greater detail below.

### VRSA strains lack a functional *SauI* restriction-modification system.

The *SauI* type I restriction-modification system has been shown to limit the uptake of foreign DNA through conjugation or transduction in S. aureus (24). In the present study, the R subunit that is responsible for restriction of foreign DNA is a pseudogene due to a C nucleotide deletion at position 1381 in all S. aureus isolates from the patient. Only an isolate from the patient’s roommate, MRSA-WC000, contains a functional R subunit, which would make it capable of restricting incoming foreign DNA.

### Understanding how VRSA isolates with very different PFGE profiles arose within a few months from a single ancestor.

PFGE analysis of VRSA isolates from the patient in the current (Table 1) and a previous study (12) showed a number of different PFGE patterns, suggesting that multiple strains of S. aureus had become independently resistant to vancomycin. However, an alternative hypothesis is that a single MRSA isolate acquired the *vanA* locus and then diverged through genome rearrangements, rather than point mutations, giving rise to the different PFGE patterns. This hypothesis is supported by the following findings.

**FIG 4:**
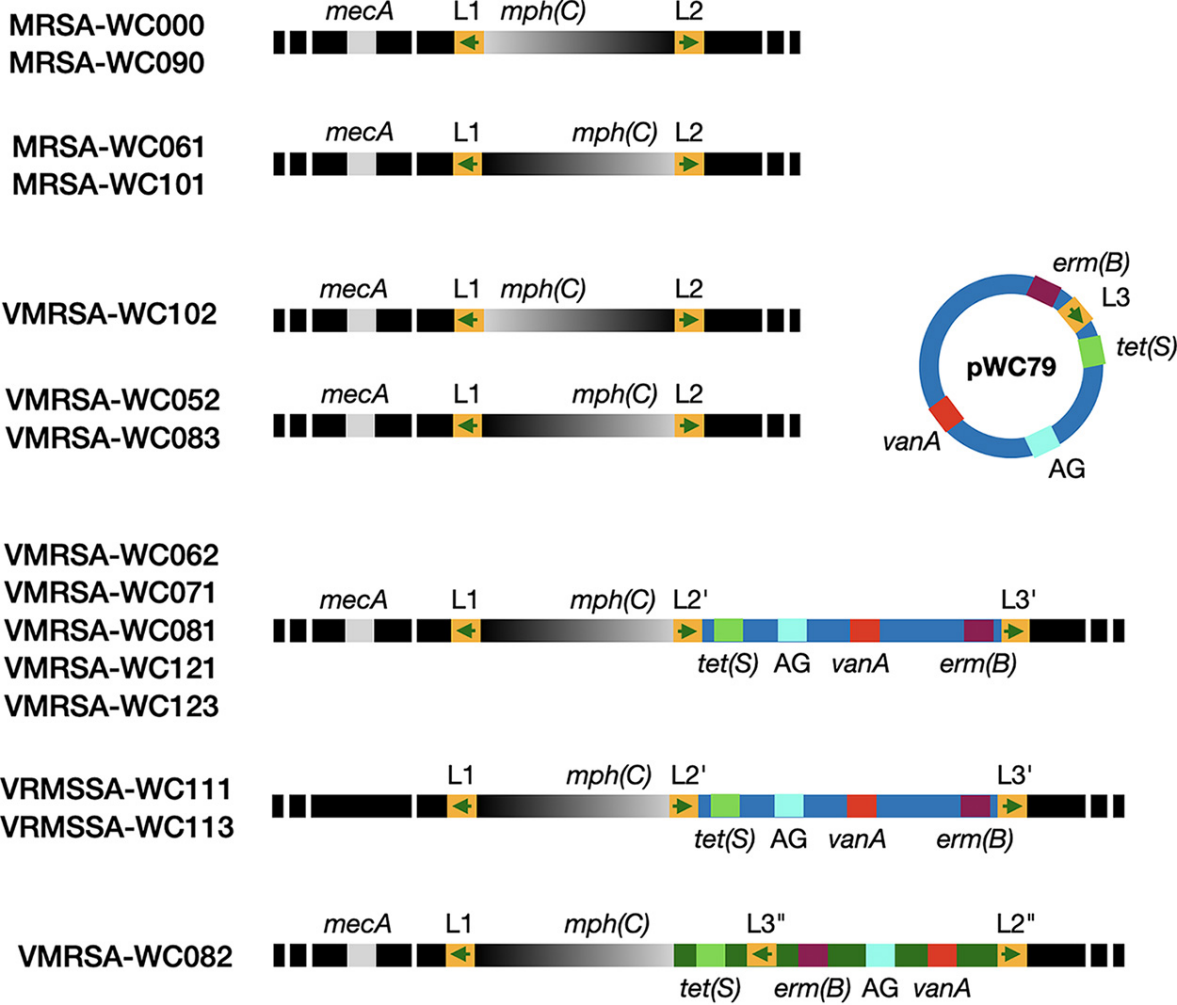
Schematic representation of the chromosome and the vancomycin resistance-conferring plasmid pWC79 in S. aureus. Sections of the chromosome are indicated by black bars with hashed ends. A gray-black gradient marks a region of the chromosome that underwent an inversion. Plasmid pWC79 is indicated by a blue background; a dark green background indicates that the plasmid has undergone significant rearrangements in strain VMRSA-WC082. Colored boxes indicate various antibiotic resistance genes. AG, aminoglycoside resistance genes. Arrows show the orientation of the *ant(6)-sat4-aph(3′)* loci L1 to L3; L2′ and L3′ indicate loci after integration of plasmid pWC79; L2″ and L3″ denote loci after undergoing further rearrangements.

First, as previously mentioned, only three isolates, VMRSA-WC052, VMRSA-WC083, and VMRSA-WC102, possess pWC79 as an independent, circular molecule, while the plasmid is integrated into the chromosome in the remaining eight VRSA isolates (Fig. 4).

Second, genome comparisons identified the inversion of entire regions that are flanked by inverted repeats. One involves Tn*4001*, a composite transposon with two IS*256* elements that form indirect repeats (20). The orientation of Tn*4001* in pWC79 is reversed in VMRSA-WC083 and VMRSA-WC102 compared to all other VRSA isolates, suggesting that either the transposon inserted into the same position in two different orientations or that it was inverted through homologous recombination between the two IS*256* indirect repeats after the insertion.

**FIG 5:**
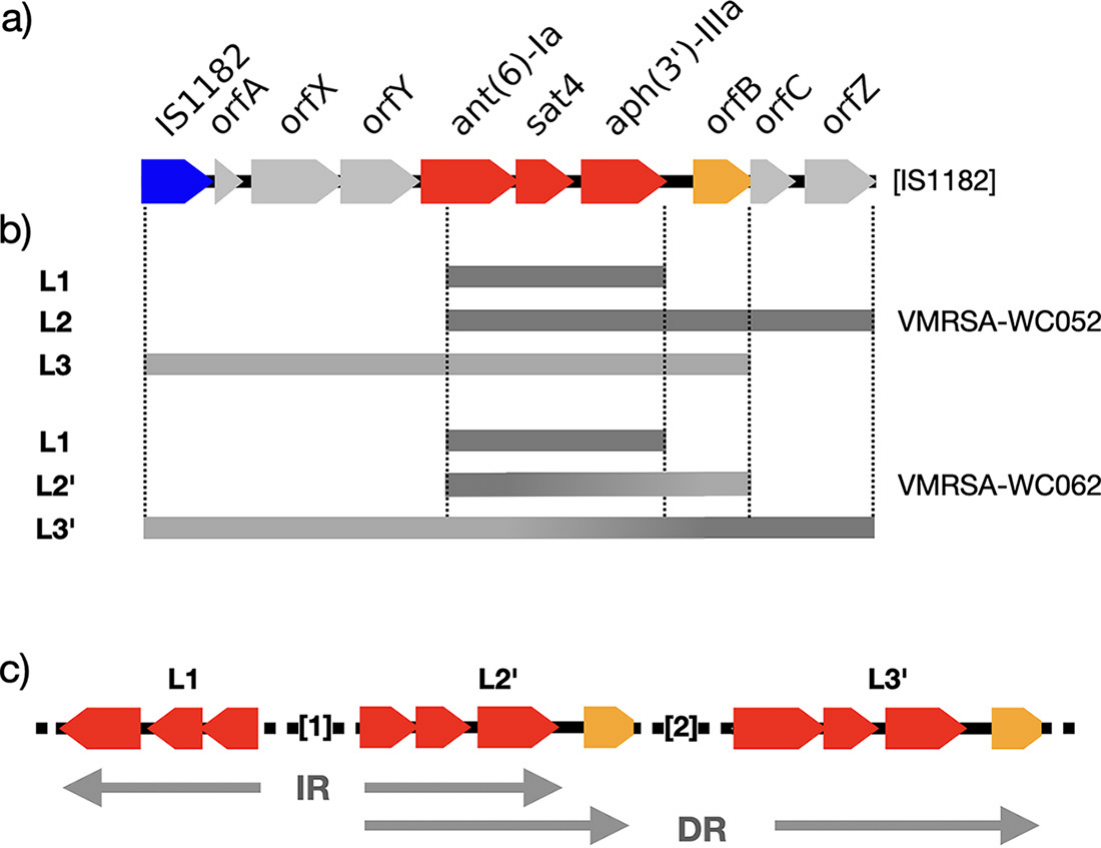
Schematic representations of transposon Tn*5405* and the *ant*(6)-*sat*4-*aph*(3′) loci in VMRSA-WC052 and VMRSA-WC062. (a) Reconstituted composite transposon Tn*5405* at the L3′ site where integration of plasmid pWC79 into the chromosome resulted in the combination of the two halves of the transposon that had been separated on the chromosome (locus L2) and the plasmid (locus L3). [IS*1182*] denotes the location of the missing second IS*1182* element to complete Tn*5405*. Open reading frames *orfX*-*orfZ* were initially described by Derbise (28–30) and encode a nucleotidyltransferase domain-containing protein, a class I SAM-dependent methyltransferase, and a RibD family protein. Open reading frames *orfA*-*orfC* encode a single-stranded DNA-binding protein, an HTH domain-containing protein, and a WYL domain-containing protein. (b) Schematic depiction of the three *ant*(6)-*sat*4-*aph*(3′) loci, L1 to L3, in VMRSA-WC052, representing a strain with a free pWC79 plasmid. While L1 remained unaffected in VMRSA-WC062, L2′ and L3′ represent the *ant*(6)-*sat*4-*aph*(3′) loci after integration of pWC79 into the chromosome. In L1 and L2/L2′, the *ant(6)-Ia* gene is truncated relative to the allele provided by the plasmid in L3/L3′. (c) Schematic representation of the three *ant*(6)-*sat*4-*aph*(3′) loci that result in an indirect repeat (IR) and a direct repeat (DR) in S. aureus isolates with chromosomal integration of the pWC79 plasmid (with the exception of VMRSA-WC082). [1], the *mph(C)*-containing region that is inverted in some of the S. aureus isolates; [2], the remaining sequence of pWC79.

Third, another inversion event involves a chromosomal region of 24 kb that includes the *msr*(A) and *mph*(C) resistance genes. The region is in the *msr*(A)-*mph*(C) orientation in all S. aureus isolates except for VMRSA-WC102, MRSA-WC000, and MRSA-WC090, where it is in the opposite orientation. The entire region is flanked by the two *ant(6)-sat4-aph(3′)* loci L1 and L2, which are oriented as indirect repeats (Fig. 4 and 5).

The fourth finding is that two of the eight isolates with a chromosomal *vanA* locus, VRMSSA-WC111 and VRMSSA-WC113, have a methicillin-susceptible phenotype and lack the SCC*mec* locus that contains the *mecA* gene. This suggests that either MRSA and MSSA isolates independently acquired the *vanA* locus or one or more VMRSA isolates lost the SCC*mec* locus after obtaining *vanA*, e.g., to compensate for the added fitness cost of becoming vancomycin resistant (25).

**FIG 6:**
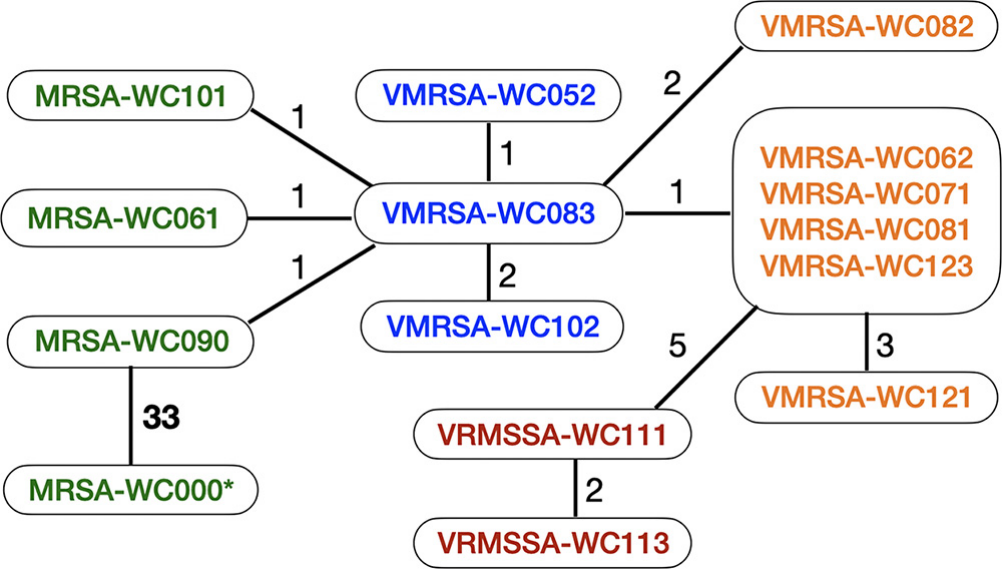
Minimum spanning tree. Illumina reads were mapped to VMRSA-WC052 as a common reference. Numbers indicate the number of single nucleotide polymorphisms (SNP) between a pair of isolates. Lines connect isolate pairs that are most similar to each other. Isolates in the same bubble have no differentiating SNPs. Green, MRSA isolates; blue, VMRSA isolates with pWC79 as a free plasmid; orange and magenta, VMRSA and VRMSSA isolates, respectively, with chromosomally integrated pWC79. An asterisk (*) denotes the isolate from the patient’s roommate.

Finally, mapping each isolate’s Illumina reads to the genome sequence of VMRSA-WC052 as a common reference shows that none of the isolates differ by more than nine single nucleotide polymorphisms (SNP) from the reference. The only exception is MRSA-WC000, which was isolated from the patient’s roommate and which differs by 33 SNPs from the closest isolate, MRSA-WC090 (Fig. 6). Most isolate pairs from the patient differ by 2 or fewer SNPs, and no pair differs by more than 11 SNPs. Even the VRMSSA isolates differ by only 5 to 7 SNPs from the nearest VMRSA isolates, which is consistent with the hypothesis that the SCC*mec* locus was lost in these isolates. VRMSSA-WC113 (collected on 24 June 2004) and VMRSA-WC121 (collected on 18 August 2004) were the most divergent isolate pair obtained from the patient, with 11 SNPs. By comparison, MRSA-WC000 from the roommate (collected 2 April 2004) and the closest isolate from the patient, MRSA-WC090 (collected 24 June 2004), differ by 33 SNPs.

The integration of pWC79, excision of SCC*mec*, and inversion of two genomic regions show how MGEs increase genome plasticity, either through transposition or by providing repeat regions for homologous recombination. This genetic mobility can explain the different PFGE patterns described earlier (Table 1) (12). The SNP analysis supports the hypothesis that a single isolate diverged after acquiring the *vanA* gene.

### Integration of pWC79 into the chromosome of S. aureus was mediated through homologous recombination between two *ant(6)-sat4-aph(3′)* loci.

The VRSA isolates described here are noteworthy because the majority contain an integrated *vanA* locus. This was of interest because all other VRSA previously documented in the United States reportedly contained a *vanA* locus that was located on a plasmid.

All VRSA isolates in this study possess three *ant(6)-sat4-aph(3′)* loci, two of which, L1 and L2, are located on the chromosome, while the third, L3, is located on plasmid pWC79. In VRSA isolates with a chromosomal *vanA* locus, the entire plasmid is integrated through homologous recombination between L2 and L3 (Fig. 4). The presence of an additional gene, *orfB*, in both L2 and L3 increases the size of homologous DNA relative to L1, which lacks *orfB* (Fig. 5).

The *ant(6)-sat4-aph(3′)* locus has been described as part of the composite transposon Tn*5405* (Fig. 5). While the chromosomal L2 locus includes genes found near the 3′ end of the transposon, locus L3 on the plasmid includes the 5′ half of Tn*5405* (Fig. 5). After integration of pWC79 into the chromosome, the combination of L2 and L3 restores the complete Tn*5405* sequence, except for a second IS*1182* that is missing at the 3′ end. Fig. 5 shows how the three *ant(6)-sat4-aph(3′)* loci are oriented relative to each other after the integration of pWC79, resulting in an inverted and a direct repeat. The inverted repeat formed by L1 and L2 is thought to have mediated the inversion of the 24 kb region containing the *msr*(A) and *mph*(C) genes in some of the isolates, while the direct repeat formed between L2 and L3 is the result of the integration of pWC79.

**FIG 7:**
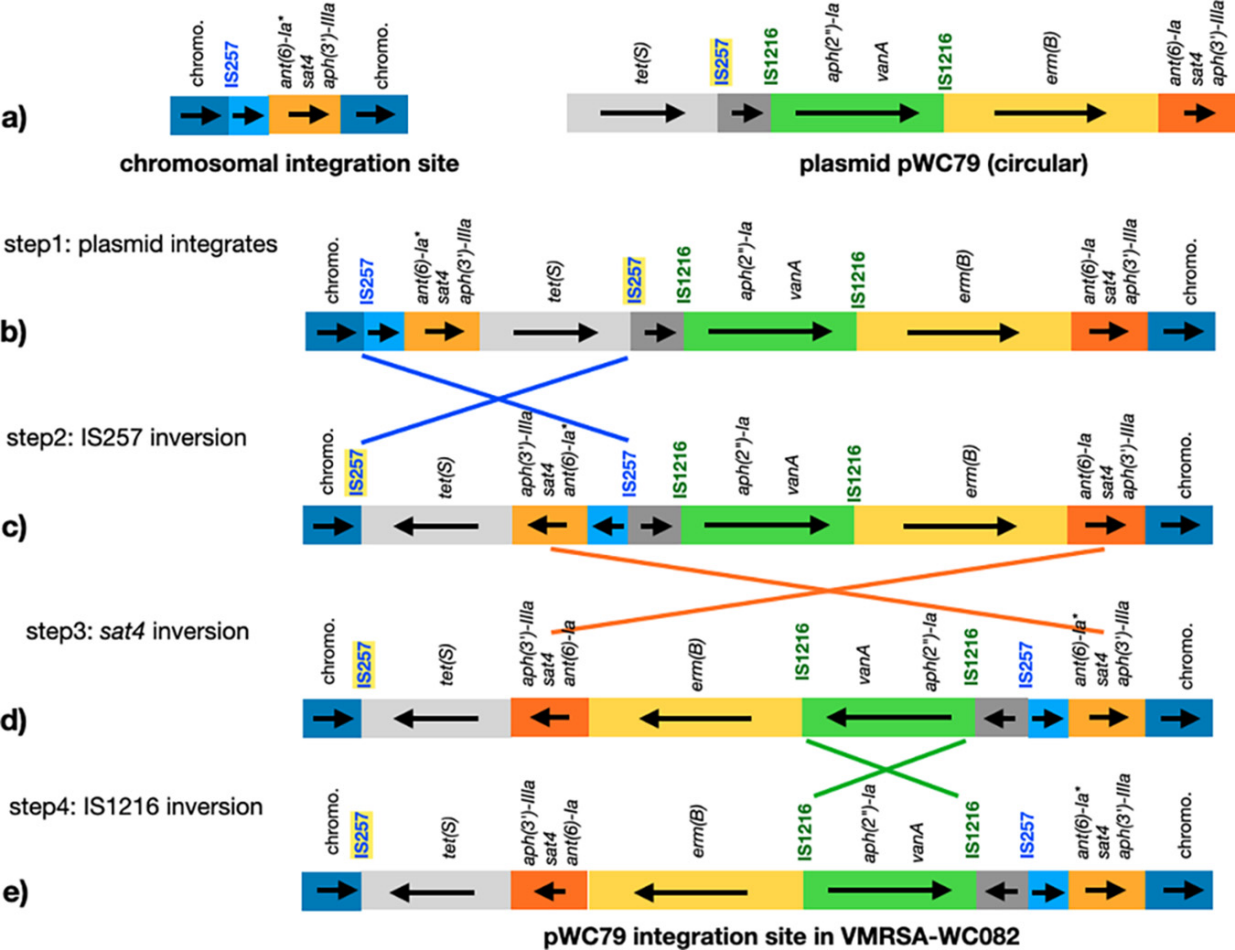
Schematic representation of how three recombination events between inverted repeats could have led to the rearrangements observed in isolate VMRSA-WC082 after integration of plasmid pWC79 into the chromosome. Isolates VMRSA-WC052, VMRSA-WC083, and VMRSA-WC102 are shown in panel a. Isolate VMRSA-WC082, after having undergone multiple inversion events, is schematically depicted in panel e. The remaining VMRSA and VRMSSA isolates, having an integrated copy of pWC79, are represented in panel b. Colored lines indicate the regions undergoing inversion. The IS*257* transposase that is unique to this isolate is highlighted in yellow. The *ant*(6)-*sat*4-*aph*(3′) loci are designated as L2/L2′ and L3/L3′, in Fig. 4. Key genes are indicated. chromo., chromosome.

Isolate VMRSA-WC082 was very distinct from the other isolates because it appears that the pWC79 plasmid underwent several recombination events after integrating into the chromosome. One feature that sets VMRSA-WC082 apart from the other isolates is an additional copy of an IS*257* transposase. Based on the model depicted in Fig. 7, an inverted repeat formed by two IS*257* elements led to the first inversion event, followed by a second inversion mediated by the two *ant(6)-sat4-aph(3′)* loci, L2′ and L3′, and a third inversion between two IS*1216* elements. The genetic evidence provided here implicates the two *ant(6)-sat4-aph(3′)* loci, as well as other repeat regions from MGEs, in large-scale DNA rearrangements.

## DISCUSSION

Antibiotic resistance, especially in S. aureus, is a significant cause of prolonged morbidity and increased mortality (26). Here, we present a detailed bacteriological and genetic analysis of 11 VRSA isolates collected from a single patient over the course of 4.5 months while residing at a long-term-care facility in New York State. At the time of collection in 2004, this was only the third time that high-level VRSA isolates had been reported in the United States, and it is, to our knowledge, the only longitudinal study (11). Two of the initial VRSA isolates from the patient, termed VRSA-3a and VRSA-3b, had been sequenced previously using either Illumina, 454 FLX, or Ion PGM system sequencing technologies (16, 17). While these short-read sequencing technologies yield valuable data, they are only able to produce fragmented genome assemblies that do not provide information on how the contigs are ordered and oriented relative to each other. Using a combination of short Illumina reads and long Oxford Nanopore Technologies (ONT) reads, we reconstructed closed, circular chromosomes and plasmids for almost all isolates. Only the assembly for isolate VRE-WC084 resulted in a circular chromosome, a circular plasmid, and two contigs of 52 and 216 kb, respectively, which might be derived from one or two plasmids.

Comparing the genomes of the various VRSA isolates showed evidence for large-scale recombination events in some isolates, including the inversion of a 24 kb region and the integration of a 79 kb plasmid, pWC79, into the chromosome. The latter is remarkable because pWC79 harbors the *vanA* locus that is responsible for high-level vancomycin resistance. This represents, to our knowledge, the first description of the *vanA* locus residing on the chromosome of S. aureus. Previous studies had noted that the Tn*1546* transposon that harbors *vanA* always resides on a plasmid in S. aureus, even though the chromosome would make a bigger target for transposition (16, 17). However, a direct transposition of Tn*1546* seems to be highly unlikely for the isolates described in the present study since the genes and sequences required for transposition, such as the left inverted repeat, the transposase, and the resolvase, are either missing from the genome or truncated. In this case, instead of transposing Tn*1546*, we provide evidence that the entire plasmid integrated into the chromosome through homologous recombination between two repeat regions. This could explain why some bacteria maintained a stable vancomycin-resistant phenotype even in the absence of antibiotic selection (12).

While the chromosomal location of *vanA* in some of the S. aureus isolates described here appears to be a first, there have been reports of plasmids similar to pWC79 integrating into the chromosome. Schwarz et al. (10) showed that plasmid pRE25, which carries the rep2 (Inc18) replicon, *erm*(B), *ant(6)-Ia*, *sat4*, and *aph(3′)-IIIa*, among other genes, transferred from enterococci to other species via conjugation and then integrated into the chromosome. From there, the plasmid excised again and transferred into a new host (10).

In the present study, pWC79 was present in all VRSA isolates, either as a free plasmid or integrated into the chromosome. However, while regions similar to those in pWC79 were also found in plasmids and contigs from VRE isolated from the same patient, none of the VRE isolates available for this study contained an exact copy of pWC79. In an earlier study that used a different set of isolates collected from the same patient, Weigel et al. reported isolating plasmid pLW2547 from E. faecium VRE2547 and plasmid pLW595 from S. aureus VRSA595 (12). Both plasmids had virtually identical BglII restriction patterns and both carried the *tet*(S) and *vanA* genes, suggesting that VRE2547 was the source of the *vanA* gene in VRSA595. Plasmid pWC79 from the current study also harbors the *tet*(S) and *vanA* genes, and the predicted BglII restriction pattern matches that of pLW2547 and pLW595 (data not shown), suggesting that pWC79 might be similar or identical to pLW2547 and pLW595. Unfortunately, neither strain VRE2547 nor VRSA595 was available for sequencing in the present study.

Comparing pWC79’s sequence to that of the plasmids present in the available VRE isolates indicates that pWC79’s origins can be traced back to mosaic plasmids that can be found in E. faecalis and E. faecium. The genes for *vanA*, *erm*(B), *ant(6)-Ia*, *sat4*, *aph(3′)-IIIa*, and *tet*(S) are present in pWC84 from E. faecium, while the *vanA* and *aph(2″)-aac(6′)* genes were found in pWC91 from E. faecalis. This finding highlights that a multispecies biofilm, such as the patient’s nephrostomy tube, can serve as a site for interspecies gene exchange.

The data presented here indicate a central role for the *ant(6)-sat4-aph(3′)* locus in the evolution of the NY VRSA isolates. Previous studies had noted the presence of these genes in the context of antibiotic resistance (12, 17). However, comparing the genomes of multiple MRSA, VMRSA, and VRMSSA isolates revealed the presence of multiple *ant(6)-sat4-aph(3′)* loci. The locus is present in two copies on the chromosome, and a third copy is located on pWC79; in combination, they serve as repeat regions for homologous recombination. One resulted in the inversion of a 24 kb region in VMRSA-WC102, MRSA-WC000, and MRSA-WC090, another facilitated the integration of pWC79 into the chromosome in some VRSA isolates, and a third resulted in an inversion that affected a part of the chromosomally integrated pWC79 in VMRSA-WC082. The *ant(6)-sat4-aph(3′)* locus has been associated with the composite transposon Tn*5405* and numerous plasmids (10, 27–31), which could explain the finding of multiple copies in one cell. Interestingly, various parts of Tn*5405* were present on the chromosome and on the plasmid, and integration of the plasmid into the chromosome nearly restored Tn*5405*, except for a missing second IS*1182* element. While the first chromosomal locus only consisted of *ant(6)-Ia*, *sat4*, and *aph(3′)-IIIa*, the other two loci had a fourth gene in common, *orfB*. This larger region of homology would better facilitate homologous recombination and might explain pWC79’s preference for the second *ant(6)-sat4-aph(3′)* locus as an insertion site.

A question of interest was to determine if multiple VRSA strains emerged independently at various time points in the patient. The presence of VRSA isolates with multiple PFGE patterns, strains with different antibiotic resistance gene content (12, 17), and the existence of methicillin-resistant and methicillin-susceptible VRSA isolates in the present study suggested that more than one S. aureus isolate could have acquired pWC79 from a VRE isolate. However, the paucity of SNPs and evidence of various recombination events suggests an alternative hypothesis. The inversion of entire genomic regions and the integration and excision of pWC79 could explain variations in PFGE patterns. The loss of the SCC*mec* locus that confers methicillin resistance would explain the simultaneous finding of VMRSA and VRMSSA isolates (32, 33). Périchon and Courvalin (34) demonstrated the incompatibility of high-level methicillin and vancomycin resistance in S. aureus. This could be the driving force for losing either the SCC*mec* or the *vanA* locus and might explain, at least in part, why some isolates have a lower than expected vancomycin MIC (Table 1). Another reason for the unexpectedly low MIC values could be that glycopeptide induction of the *vanA* locus exerts a high fitness cost on VRSA isolates. (35) This was shown to result in an extended lag phase and slower rate of exponential growth, which could give the appearance that the bacteria are not highly resistant when measured by the standard Etest.

Cells that contain plasmids with antibiotic resistance genes often pay a fitness cost in the absence of antibiotic selection (36–38), which allows faster-growing, plasmid-free cells to expand within the population. During periods of antibiotic selection, resistant cells can then rapidly replace the susceptible cells that were killed by the antibiotic. This repeated selection for different subpopulations would explain why VMRSA, VRMSSA, and MRSA were isolated from the same patient over a period of time and why these isolates differ by only a few SNPs as described below.

The results of the SNP analysis indicate that the two most divergent S. aureus isolates from the patient, a VMRSA and a VRMSSA strain, differ by only 11 SNPs. By comparison, MRSA isolates from the patient and a MRSA isolate from the roommate differ by 33 or more SNPs. These results indicate that only one isolate obtained the *vanA* gene from a coinfecting VRE isolate and then diverged through various recombination events that include inversions of the genome, loss of SCC*mec*, and the integration of the *vanA* plasmid pWC79 into the chromosome. Interestingly, while the roommate’s MRSA isolate contained a functional *SauI* type I restriction-modification system, the VRSA isolates from the patient had apparently lost that ability due to a mutation in the R subunit. If and how this could have facilitated the horizontal gene transfer of pWC79 into these isolates remains to be determined.

While colonization with MRSA and VRE strains is not uncommon among long-term care patients (39) and those with indwelling medical devices, it is surprising that more VRSA strains have not been detected in the United States. Having multiple VRSA isolates from the same patient that are spread out over several months provided a unique opportunity for comparisons and an improved understanding of the microbiology and the potential for *vanA* plasmids to incorporate into the genomes of S. aureus strains. WGS analysis combining long and short sequencing reads provided a better understanding of the development and maintenance of VRSA from this NY patient and elucidated the genetics of these antibiotic-resistant organisms. Continued vigilance detecting antibiotic resistance and assessing transmission through infection control efforts as described in this investigation will positively impact public health in the future.

## MATERIALS AND METHODS

### Bacterial isolates.

Following the initial isolation of a VRSA isolate from a urine culture in March of 2004, numerous specimens were collected during the subsequent months from the patient, her roommate of many years, and nursing home staff members. Clinical isolates were cultured on Trypticase soy agar plates with 5% sheep’s blood (Wadsworth Center Tissue and Media Core). Plates were examined for colonies resembling S. aureus; identification of S. aureus was confirmed by Gram stain, catalase, coagulase, and *nuc* gene detection by real-time PCR. Methicillin and vancomycin resistance was confirmed by MIC testing with oxacillin and vancomycin Etest (AB Biodisk, Solna, Sweden), according to interpretive criteria of the Clinical and Laboratory Standards Institute (CLSI). Vancomycin resistance was confirmed by Etest after revival of isolates from freezer stocks. Real-time PCR was used to screen for the *vanA*, *vanB*, v*anD*, and *mec*A genes. For pulsed-field gel electrophoresis (PFGE) analysis, genomic DNA was digested with SmaI restriction endonuclease, DNA fragments were separated, and PFGE patterns were analyzed and compared using the BioNumerics software package (Applied Math, Kortrijk, Belgium).

### Illumina short-read sequencing.

Single colonies were selected for sequencing based on the date of isolation, antibiotic resistance profile, PFGE pattern, and colony morphology to get a broad spectrum of isolates. Isolates were subcultured from frozen stocks twice on Trypticase soy agar with 5% sheep’s blood and incubated overnight at 37°C. For genomic DNA isolation, samples were preprocessed by inoculating a 1-μL loopful of growth from an isolated colony into 180 μL enzymatic lysis buffer (1 M Tris-HCl, pH 8.0, 0.5 M EDTA, and Triton X-100) and 1 μL of lysostaphin (1 mg/mL). Samples were incubated at 56°C for 30 min at 350 rpm in a MultiTherm shaker (Benchmark Scientific), followed by the addition of 25 μL of proteinase K (Qiagen) and a second incubation for 30 min at 56°C. Finally, 4 μL of RNase A (100 mg/mL) was added to each sample and incubated at room temperature for 3 min. All samples were then processed using the DNeasy blood and tissue kit (Qiagen) according to the manufacturer’s instructions. Nucleic acid was eluted into 10 mM Tris, quantitated using the Qubit double-stranded DNA (dsDNA) high-sensitivity (HS) assay kit (Life Technologies) on a Qubit 4.0 fluorometer (Life Technologies), and submitted to the WC Applied Genomics Technologies Core for WGS using the Illumina MiSeq platform. The Nextera XT protocol (Illumina) was used to generate sample libraries that produced paired-end reads of 250 bp in size as described previously (40).

### Oxford Nanopore long-read sequencing.

Isolates were subcultured from frozen stocks twice on Trypticase soy agar with 5% sheep’s blood. For genomic DNA extraction, colonies were resuspended in 1.5 mL sterile water to a McFarland concentration of 3.0 and harvested as pellets. The pellets were resuspended in 100 μL STET buffer (8% sucrose, 50 mM Tris-HCl, pH 8.0, 50 mM EDTA, pH 8.0, 5% Triton X-100), 5 μL lysozyme (100 mg/mL, Sigma-Aldrich), and 10 μL lysostaphin (5 mg/mL, Sigma-Aldrich). High-molecular-weight genomic DNA was extracted using the Nanobind CBB Big DNA kit (Circulomics) following the Gram-positive extraction protocol. Genomic DNA was quantified using a Qubit 4.0 fluorometer (Thermo Fisher Scientific). The quality of the genomic DNA was assessed using the TapeStation system (Agilent).

MinION sequencing libraries were prepared from 1.5 μg of input DNA. Genomic DNA was sheared in 50 μL total volume in Covaris G tubes using an Eppendorf 5425 centrifuge at 6,000 rpm. Sequencing libraries were prepared according to the manufacturer’s instructions (Oxford Nanopore) and multiplexed using 1D Native barcoding kits (EXP-NBD104, EXP-NBD114) followed by a ligation and sequencing kit (SQK-LSK109). The library was loaded on a SpotON flow cell R9.4.1 FLO-MIN106 and sequenced for 72 h on the MinION device. The fast5 data from MinKNOW (version 20.10.6) was converted to fastq format using the Guppy base caller (version 4.2.3) in fast mode on a MinIT device (Oxford Nanopore, United Kingdom).

### Bioinformatic analyses.

Paired and unpaired Illumina reads were trimmed with Trimmomatic (version 0.39, parameters: ILLUMINACLIP:NexteraPE-PE.fa:2:30:10 LEADING:3 TRAILING:3 SLIDINGWINDOW:4:20 MINLEN:100) (41), while Oxford Nanopore Technologies (ONT) reads were trimmed with Porechop (version 0.2.4) (42). Hybrid *de novo* assembly of genomes was done with Unicycler (version 0.4.8) (43), which included Racon (version 1.3.1) to polish hybrid assemblies and several rounds of Pilon (version 1.22) to repair any small-scale errors. Unicycler was run in all three available assembly modes (conservative, normal, bold); the mode providing the best result for each isolate is shown in Table 2.

As quality control, reads were mapped to the assembly created by Unicycler using BWA MEM (44, 45). Default options were used for the Illumina reads, while option “-x ont2d” was used for the ONT reads. The resulting BAM files were analyzed with Pilon (46), whose output was visualized with the Integrative Genome Browser (version 9.1.10) (47) to identify regions of concern. DNA fragments were identified as assembly artifacts and removed from the genome if they contained any regions of zero coverage according to the corresponding BAM file. These fragments were always of very low coverage, less than 5 kb in size, and duplications of chromosomal regions, and all but one were linear molecules. At most, six artifacts, totaling 8.7 kb, were removed from an assembly, although most were free of artifacts.

PlasmidFinder (version 2.1) was used to detect plasmid replicon sequences for Gram-positive bacteria in all genomes (48). Genomes were annotated with the stand-alone version of NCBI’s Prokaryotic Genome Annotation Pipeline (PGAP) (49). Where necessary, circular DNA molecules were rearranged to begin either with the *dnaA* gene or a plasmid replication initiation protein. ABRicate, using the NCBI database, was used to find antibiotic resistance genes (50, 51). In cases of discrepancies between the PGAP and ABRicate annotations, the PGAP annotations were used.

The program progressiveMauve was used for genome sequence comparisons (22). For single nucleotide polymorphism (SNP) analysis, Illumina sequence reads from MRSA and VRSA isolates were mapped with BWA to reference VMRSA-WC052 to perform a SNP analysis with FreeBayes (27), which is part of the LegioCluster pipeline (52). The analysis included plasmids and other mobile genetic elements; however, questionable or low-quality SNP calls were removed with vcffilter (53) if the SNP quality assigned by FreeBayes was less than 20, the read depth was less than 10, the alternate allele quality sum was less than 20, the count of full observations of an alternate haplotype exceeded 10% of all counts, or the reads supporting a SNP call were limited to only one of two strands.

### Data availability.

The genomes are available at NCBI as follows: VRE-WC031: CP092576 to CP092580, VSE-WC032: CP092574 to CP092575, VRE-WC072: CP092569 to CP092573, VRE-WC084: CP092583 to CP092586, MRSA-WC000: CP092567 to CP092568, MRSA-WC061: CP092565 to CP092566, MRSA-WC090: CP092563 to CP092564, MRSA-WC101: CP092561 to CP092562, VMRSA-WC052: CP092558 to CP092560, VMRSA-WC062: CP092556 to CP092557, VMRSA-WC071: CP092554 to CP092555, VMRSA-WC081: CP092552 to CP092553, VMRSA-WC082: CP092550 to CP092551, VMRSA-WC083: CP092547 to CP092549, VMRSA-WC102: CP092544 to CP092546, VRMSSA-WC111: CP092581 to CP092582, VRMSSA-WC113: CP092542 to CP092543, VMRSA-WC121: CP092540 to CP092541, VMRSA-WC123: CP092538 to CP092539. BioProject: PRJNA937246.
